# Old Habits Die Hard: Dietary Habits of Migraine Patients Challenge our Understanding of Dietary Triggers

**DOI:** 10.3389/fneur.2021.748419

**Published:** 2021-11-18

**Authors:** Marco Lisicki, Jean Schoenen

**Affiliations:** ^1^Neuroscience Unit, Conci·Carpinella Institute, Córdoba, Argentina; ^2^Headache Research Unit, Department of Neurology, CHR Citadelle, University of Liège, Liège, Belgium

**Keywords:** headache, diet, metabolism, survey, prospective

## Abstract

**Introduction:** Migraine is a multifactorial neurological disorder with a major metabolic facet. Dietary approaches represent a commonly implemented lifestyle modifying strategy in headache clinics, yet the precise relationship between diet and migraine is still a matter of debate.

**Materials and Methods:** The study consisted of two parts: first, in a cross-sectional design, we compared alimentary habits of migraine subjects and a control group of healthy volunteers. For the second part, we prospectively evaluated patients' daily consumption of various potentially migraine-triggering foods over a two-month period in order to examine their possible association with the occurrence of a migraine attack.

**Results:** Most migraine patients reported avoiding at least one potentially migraine-triggering food/drink from their diet. In spite of that, with the sole exemption of citrus fruits, there were no statistically significant differences with respect to consumption patterns between migraine patients and controls (including wine and chocolate). Consumption frequency over time was proportional to intake of potentially migraine-triggering foods the day before a migraine attack.

**Conclusion:** Our results underline the need of performing trigger challenges in order to avoid falling into an association-causation fallacy when attempting to identify possible alimentary migraine triggers. Indeed, it is possible that intake of certain foods like chocolate before attacks is a consequence of pre-attack cravings or a simple coincidence facilitated by previously established dietary habits.

## Introduction

Migraine is a multifactorial neurological disorder with a major metabolic facet (1). While the genetically determined aspects of this disease cannot be modified, much can be done with respect to environmental factors in order to improve migraine care (2). Lifestyle modifying strategies become progressively more refined as their supporting scientific evidence grows, allowing physicians to offer patients more efficacious, non-pharmacological, therapeutic approaches, which represents a highly increasing demand in headache clinics.

The possible relation between diet and migraine has been the subject of numerous debates in the past. Migraine patients often recognize certain dietary factors as potential attack triggers, among which fasting, alcohol or chocolate intake are frequently mentioned (3–7). Previous reviews reported that 12 to 60% of subjects allude to foods as triggers for migraine attacks, with many of them listing more than one dietary trigger (8). However, several foods have been categorized as migraine triggers on the sole basis of a self-reported temporal association between intake and attack occurrence, based on data from cross-sectional surveys. Such designs entail a high risk of association-causation fallacy, and can be largely affected by recall bias. By the same token, scientific evidence is lacking for general recommendations on trigger avoidance to migraine patients (8). For instance, published data on the effect of elimination of specific foods, like chocolate, are contradictory (9). Studying the migraine-diet relationship is further complicated by the fact that it might be bidirectional, since having migraine could affect dietary choices (10).

Because of these problems and the natural fluctuations of migraine, (11) a prolonged prospective observation of alimentary patterns, cravings, and fasting in a ‘real-world' scenario comparing migraine patients and healthy controls might help to understand the dietary role in attack generation, and possibly pave the way for increasing knowledge of migraine pathophysiology and for longitudinal, randomized controlled trials of food elimination strategies.

We conducted a two-step study that first compared consumption and avoidance of foods between episodic migraine patients and controls, whereafter migraineurs were asked to prospectively monitor changes in appetite and consumption of various foods the day before and hours before occurrence of their migraine attacks for 2 months. Our main goal was to evaluate the migraine triggering potential of certain alimentary products and their influence of eating habits among migraine sufferers.

## Subjects and Methods

We evaluated alimentary habits in a cross-sectional survey of migraine subjects and a control group of healthy volunteers. In addition, we assessed daily consumption of various potentially migraine-triggering foods in the group of migraineurs over a 2-month period in order to examine their relationship with the occurrence of a migraine attack.

### Survey of Alimentary Habits

A questionnaire was distributed to 237 episodic migraineurs recruited among hospital and university personal (*n* = 106), patients consulting our headache clinic (*n* = 90) and students or acquaintances diagnosed with migraine (*n* = 41). The questionnaire comprised a first set of questions aimed at collecting the subjects' demographics and confirming the diagnosis of migraine and occurrence of visual auras based on the diagnostic criteria of the International Headache Society (ICHD3 1.1 or 1.2.1) 0.12), followed by a second set of questions where subjects were asked to list which foods or drinks they had avoided or eliminated because of being considered as potential triggers of their attacks (if any). Afterwards, they had to indicate their personal consumption frequency (never, ≥ 1x/month, ≥ 1x/week, every day) of the following foods and drinks: wine, beer, other alcoholic beverages, fermented cheese, citrus fruits, chocolate and sweeteners. Finally, subjects were asked to report their usual consumption of caffeinated (coffee, energetic drinks) and sweetener-containing soft drinks: how many cups, glasses, or cans per day, per week or per month, or never? The questionnaire was designed based on the available scientific evidence related to our working hypothesis. It was delivered to participants by hand or *via* mail.

A similar questionnaire, but limited only to the second part (eating habits), was submitted to 88 age- and gender- matched healthy controls, recruited among students, acquaintances and hospital staff who were not suffering from any recurrent headache.

### Migraine Attack-Related Food Consumption and Appetite Changes

For the prospective analysis, all 106 enrolled migraine patients received a two-month paper calendar in which they were instructed to register whether they had consumed a certain food or drink from the pre-specified list used in the above described survey of alimentary habits and if they had cravings or loss of appetite at the same time points the day before or hours before their migraine attacks. Missing a meal was also recorded.

### Data Analysis and Statistics

Epi Info™ 2000 for Windows (https://www.cdc.gov/epiinfo) was used for the statistical analyses of results. Proportions were compared using the Chi-square test. A *p*-value < 0.05 was considered statistically significant.

The study protocol was approved by the ethics committee of the University of Liège University Hospital.

## Results

### Subjects' Disposition and Demographics

Fifty-nine migraine patients (25%) adequately filled in the questionnaire on their eating habits: 25 (42%) from the students and acquaintances group, 19 (32%) from the hospital and university personal, and 15 (26%) from the clinic-based group. According to the answers given in the questionnaire, a diagnosis of episodic migraine without aura (code 1.1) (63%) or of migraine with aura (code 1.2.1) (37%) was confirmed in all of them.

Fifty-one migraineurs (22%) reported prospectively their appetite changes and food/drinks consumption in relation to migraine attacks for the pre-specified 2-month period. In this group, mean frequency of attacks over the 2 months was 6 (range 1–13).

Data of 77 healthy controls (87.5%) were available for analysis.

There were no differences between the migraine and control groups with respect to age or gender proportions (40 ± 11.3 vs. 41± 11.2 years; *p* = 0.61, and 91,5 vs. 92,2% females; *p* = 0.89, respectively) (Table 1).

**Table 1:** Demographic characteristics of the study sample.

	**Migraine**	**Controls**	***p*-value**
*n*	59	77	
Age	40 (±11.3)	41 (±11.2)	0.61
Female %	91,5%	92,2%	0.89
Aura^*^	37%		

* *Probable aura based on questionnaire responses*.

### Alimentary Habits

**Figure 1:**
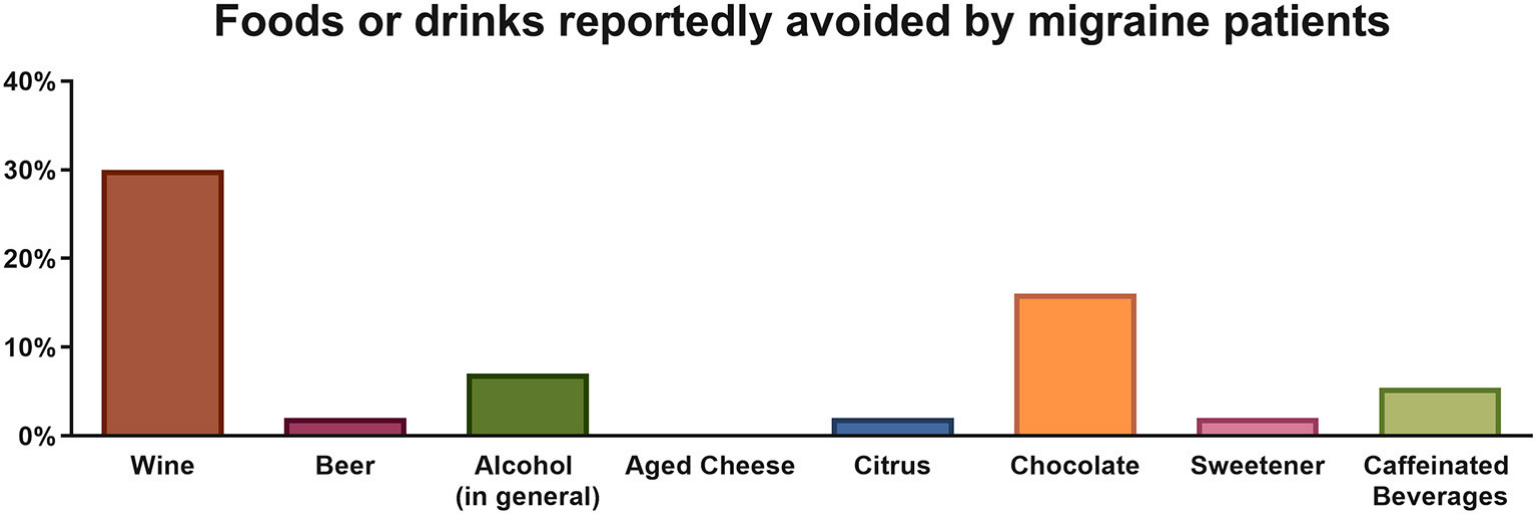
Percentage of migraine patients reportedly avoiding a food or drink commonly perceived as a trigger.

**Figure 2:**
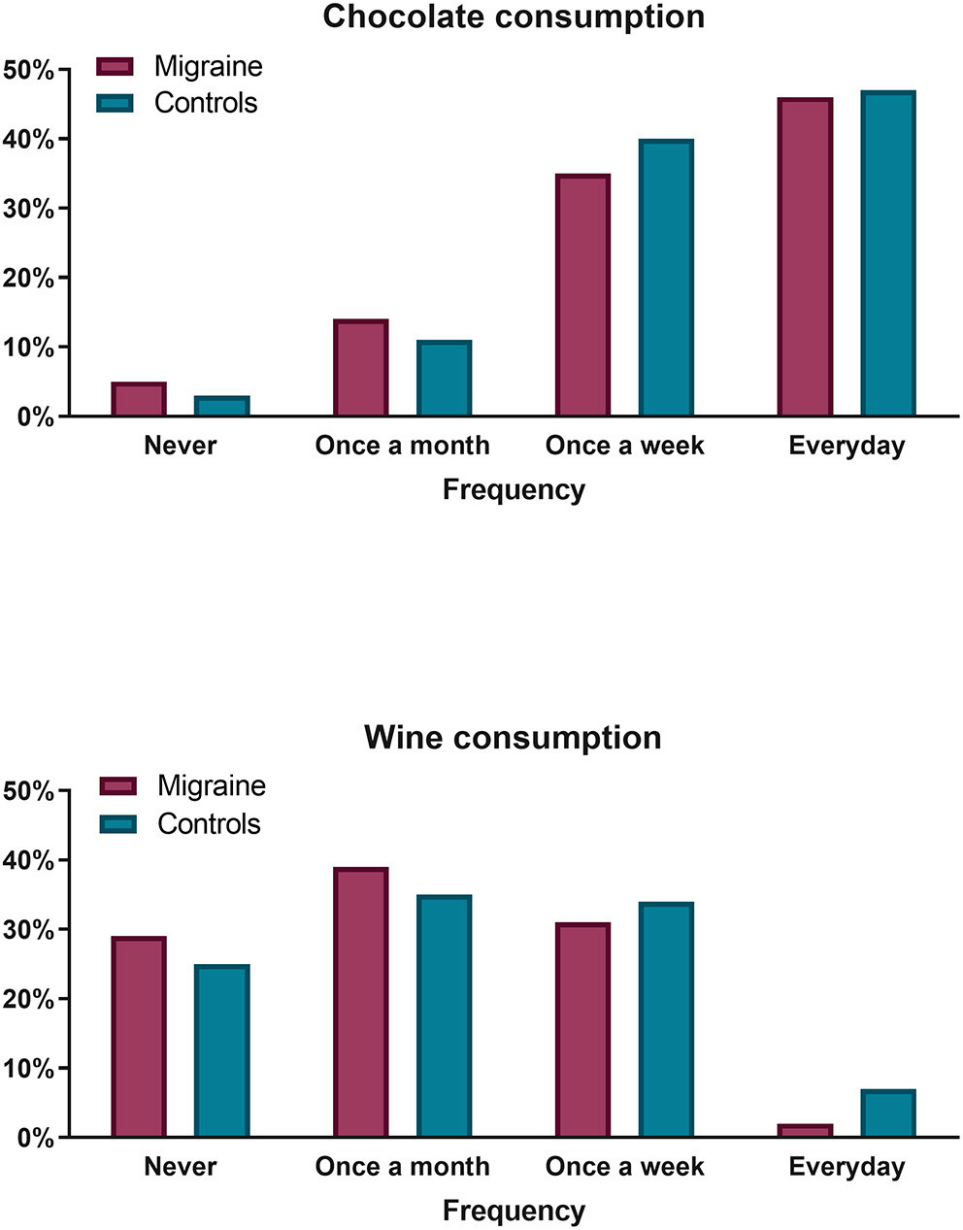
Chocolate (**upper panel**) and wine (**lower panel**) consumption frequency comparison between migraine patients (**purple bars**) and controls (**blue bars**). No significant differences were observed.

Most (64.29%) migraine patients reported avoiding at least one of the listed foods/drinks from their diet. According to the survey, the most commonly reported avoided triggers were wine (30%) and chocolate (16%) (Figure 1). In spite of that, frequency-consumption analysis revealed that there were no statistically significant differences with respect to wine, beer, alcohol in general, aged cheese, sweetener or chocolate consumption patterns between migraine and control subjects (all *p* > 0.05). For instance, consumption of wine was reported at a frequency of ≥ 1/month by 71% of migraineurs, as compared to 75% of controls, and similarly occurred for consumption at a frequency of ≥ 1/week (30.5 and 33.8% respectively). Everyday intake of chocolate was observed in 46% of migraineurs vs. 47% of controls, while only 5 and 3% respectively reported never eating chocolate (Figure 2). Twenty-five percent of migraineurs reported drinking ≥ 6 cups of coffee per day, compared to 22% of controls.

The only statistically significant intake difference observed between the two groups was in the consumption of citrus fruits, which was higher in controls (*p* = 0.019).

On the basis of these results, we specifically compared consumption frequency between migraine patients who reported having completely eliminated wine (*n* = 17) or chocolate (*n* = 8) from their diet and those who did not. We found no significant difference between these two groups regarding chocolate consumption (*p* = 0.54), but wine intake was significantly higher in the group of patients who allegedly avoid it (*p* = 0.01). In fact, only one out of the seventeen migraine subjects who reported complete elimination of wine out of their diet genuinely did not consume it.

### Migraine Attack-Related Food Consumption and Appetite Changes

Loss of appetite in at least 1 out of 4 attacks was reported by 30% of migraine subjects the day before an attack, and by 41% hours before an attack. Frequent anorexia occurring in at least 3 of 4 attacks was observed in 14% of migraineurs the day before and in 25% during the hours preceding an attack.

In at least 1 out of 4 attacks, food craving was reported by 38% of migraine subjects the day before an attack, and by 26% during the hours immediately preceding an attack. Frequent cravings (present in at least 3 of 4 attacks) were seldom observed (10% the day before and 8% hours before the attack).

### Food Consumption the day Before an Attack

The most frequently mentioned foods consumed the day before an attack in at least 1 out of 4 attacks were chocolate (*n* = 31 subjects, 17 in 3 of 4 attacks), sweeteners (*n* = 16), wine (*n* = 15), fermented cheese (*n* = 15), and citrus fruit (*n* = 13).

Weaning of coffee the day before or hours before an attack in ≥ 50% of attacks was mentioned by only 3 migraineurs consuming ≥ 3 cups of coffee per day.

**Figure 3:**
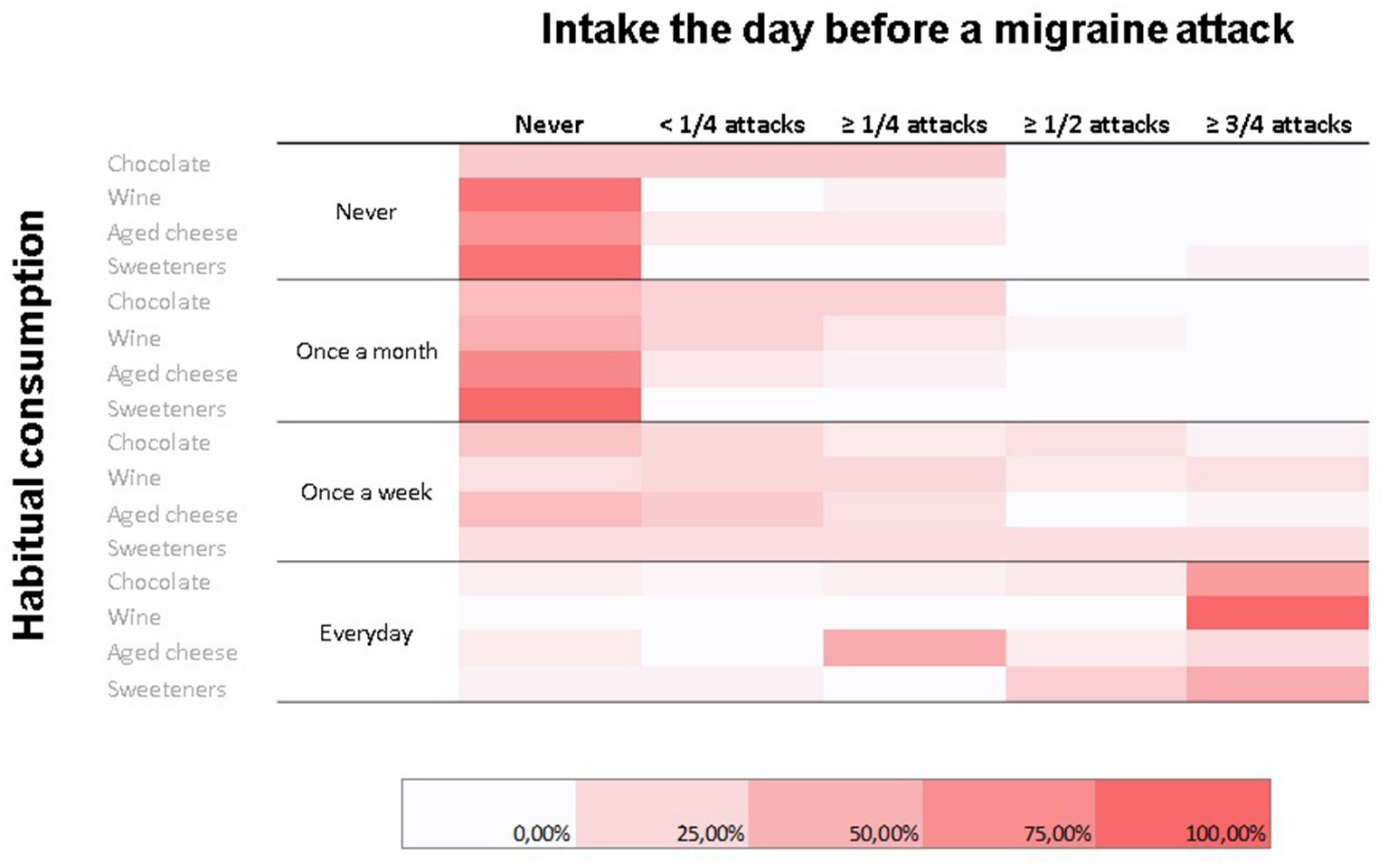
Heatmap representing relative frequencies (color-coded; color scale on the bottom) of habitual consumption habits for commonly perceived migraine triggers (**rows**) in relation to consumption the day before a migraine attack (**columns**). A statistically significant association between habitual consumption and consumption the day before a migraine attack was observed.

Not surprisingly, we found a significant association between the consumption frequency of the various foods and the percentage of attacks preceded the day before by their intake (*p* = 0.004) (Figure 3).

Intake of chocolate or wine the day before an attack was significantly more frequent in migraineurs who declared avoidance of these foods compared to those who did not (*p* = 0.015 for chocolate, *p* = 0.0001 for wine).

Finally, there was a significant association between the proportion of subjects with food craving and the proportion of those who reported chocolate consumption the day preceding an attack (*p* = 0.001).

### Food Consumption Hours Before an Attack

Regarding the time period immediately preceding an attack, the most frequently consumed foods (in at least 1 out of 4 attacks) were chocolate (*n* = 26; 11 in 3 of 4 attacks), sweeteners (*n* = 12), fermented cheese (*n* = 10) and wine (*n* = 9). Consumption of chocolate and wine hours before an attack was more frequently reported by migraineurs who declared in the initial survey that they were avoiding these foods (*p* = 0.009 for chocolate, *p* = 0.01 for wine).

Again, craving hours before an attack was associated with chocolate intake (*p* = 0.0004).

Notably, only 6 migraineurs reported that missing a meal preceded an attack in at least 1 out of 4 attacks. Caffeine withdrawal in at least 1 of 4 attacks occurred the day before an attack in only 6 subjects, but in 12 immediately before an attack.

## Discussion

In the survey of dietary habits, we found no differences between migraine patients and controls regarding frequency of consumption of various foods classically considered to be able to trigger migraine attacks (8). The only exception is intake of citrus fruits which is significantly lower in migraine sufferers than in healthy controls. To our knowledge, this has not been reported previously, has no proven physiological explanation, and needs to be verified in a larger study. Nonetheless, the majority of migraineurs (62.5%) avoid at least one food or drink because of its attack-triggering potential. This prevalence is higher than those reported in similar studies (10–45%) (12). The most frequently avoided foods in our survey were chocolate and alcoholic beverages; avoidance of wine (*n* = 17), beer (*n* = 1), and other alcoholic drinks (*n* = 4) combined is reported by 37% of migraineurs, which is within the range of 20 to 52% published in the literature (12). Interestingly, the subjects who reported to have completely eliminated wine (*n* = 17) out of their diet are also those who drink it most frequently according to the survey; only one amongst them effectively did not consume it. Contrary to wine, consumption of chocolate was not significantly higher in subjects reporting its elimination from their diet, but only 1 of these 8 subjects effectively reported zero chocolate consumption. These apparent contradictions could be due to the fact that volume of intake was not assessed by the questionnaire and hence subjects who pretend avoiding wine or chocolate may reduce quantity of intake, but not completely avoid them. There might be another explanation, in line with the recently emphasized (10) bidirectional relationship that may exist between migraine and diet. Migraine is indeed known to be comorbid with anxiety (13, 14). Migraineurs may thus not refrain from drinking alcoholic beverages or eating chocolate in moderate quantities because they get benefit from the anxiolytic properties (15, 16). This hypothesis needs to be confirmed in an adequately designed study.

Regarding the prospective monitoring of food intake in relation to migraine attacks, the first notable finding concerns the change in appetite. Cravings for sweets (or other foods) is a well-known symptom of the premonitory phase of the migraine attack (17). At whatever frequency, it is numerically more often reported the day before an attack (41%) than hours before (31%), but it occurs regularly so (3 out of 4 attacks) in only 10 and 8% respectively. By contrast, loss of appetite seems numerically more frequent hours before an attack (51%), when it is clearly more prevalent than craving, than the day before (43%).

It comes of no surprise that the most frequently consumed foods and drinks are also the most often mentioned as having been taken the day before or immediately before an attack. For instance, while 26 of 59 migraineurs declare eating chocolate every day, 17 did so the day before and 11 immediately before an attack in three out of four attacks. Avoiders of wine are also those whom most often mentioned drinking wine before an attack, which could indicate that their attacks are triggered by wine. This relationship, however, is likely biased by the fact that these migraineurs are also the most frequent consumers of wine.

If one focuses on subjects who consume wine no more than once per month, only one subject actually had a pre-attack intake of wine in half of his attacks. If a once per week consumption is considered, this figure raises up to one out of three subjects for wine and one out of four for chocolate. Whether this indicates a genuine triggering effect of these foods cannot be established without dietary elimination or proper blinded provocation studies in these subjects. As far as chocolate is concerned, its role as a trigger of attacks is not supported by our finding that pre-attack craving is highly correlated with its consumption before an attack, which merely suggests that this is part of the premonitory symptoms of the migraine attack.

Our study has several limitations. Because of the relatively poor response rate, the sample size is modest and that the majority of migraine subjects (74%) were recruited in the general population compared to only 26% amongst clinic-based patients may have biased the responses. On the other hand, studies based solely on patients consulting a headache center may obviously also be biased due to the selection of the most severely disabled patients. The questionnaire based on ICHD criteria (18) used to diagnose migraine in the non-clinic cohort was used in other studies and has acceptable sensitivity and specificity for migraine without aura. Unfortunately, diagnosing migraine aura with a questionnaire is much more difficult and less accurate. This may explain the high proportion of migraine with aura diagnoses (37%) in this study, as compared to studies using face-to-face interviews. The latter was of course the diagnostic method in the clinic-based sample of migraine patients who were all diagnosed by a headache neurologist. The retrospective survey on alimentary habits and food avoidance could have been partly influenced by recall bias. By contrast, participation rate and compliance during the prospective study on food intake in relation to an attack, a study design rarely used up to now, were excellent. For practical reasons, we had to make a selection among foods and beverages to be investigated. We chose those that are generally mentioned in the literature (see 8 for review) and, if other potential dietary triggers have been omitted, they are likely very rare and related to individual susceptibilities. One possible exemption could be watermelon, (19) but this possibility still needs to be corroborated. In addition, it has to be considered that the study was carried out in the French-speaking region of Belgium (Wallonia) and alimentary habits may vary across different cultures. Furthermore, a limited number of possible confounders were considered in this observational study and thus other factors interfering in the relationship between food intake and migraine may have been overlooked. For instance, other migraine triggering factors such as sleep deprivation or stress can also modify food consumption patterns interfering with the evaluation of a substance's attack triggering potential. Finally, although we collected data on frequency of dietary intakes, we have no information on the quantity or concentration of the various foods and drinks mentioned by patients. As mentioned, this may in part explain the discrepancy between the declaration on certain foods that are avoided and their actual frequency of intake, because “avoidance” could mean “lower quantity” or “lower frequency” in the patients' mind.

To conclude, there is overall no significant difference between migraine sufferers and healthy controls with regards to the consumption of various foods or drinks that have been previously implicated in triggering migraine-attack. The prospective recording of food consumption in relation to migraine attacks shows that chocolate, wine, sweeteners and fermented cheese are often consumed before an attack. These foods, however, cannot be considered as “attack triggers” without reservation, since they are also frequently consumed by migraine subjects, so that the temporal relation with an attack may be fortuitous. Chocolate consumption is more likely a consequence of pre-attack craving and other dietary habits may be a consequence of migraine rather than a cause of it.

Adequately designed dietary provocation studies are needed to determine if foods or drinks frequently or consistently trigger an attack. Such studies are, however, complicated by the well-known fact the migraine threshold fluctuates over time. Consequently, certain triggers may ignite an attack at some timepoints, i.e. during menses, but not at others, i.e. at mid-cycle.

## Data Availability Statement

The raw data supporting the conclusions of this article will be made available by the authors, without undue reservation.

## Ethics Statement

The study protocol was approved by the Ethics Committee of the University of Liège University Hospital. The patients/participants provided their written informed consent to participate in this study.

## Author Contributions

ML participated in data analysis and manuscript preparation. JS conceived the study participated in data analysis, and manuscript preparation. All authors contributed to the article and approved the submitted version.

## Funding

This study was supported by the University of Liège.

## Conflict of Interest

The authors declare that the research was conducted in the absence of any commercial or financial relationships that could be construed as a potential conflict of interest.

## Publisher's Note

All claims expressed in this article are solely those of the authors and do not necessarily represent those of their affiliated organizations, or those of the publisher, the editors and the reviewers. Any product that may be evaluated in this article, or claim that may be made by its manufacturer, is not guaranteed or endorsed by the publisher.
